# Human Papillomavirus and Head and Neck Cancer: Psychosocial Impact in Patients and Knowledge of the Link – A Systematic Review

**DOI:** 10.1016/j.clon.2016.02.012

**Published:** 2016-07

**Authors:** R.H. Dodd, J. Waller, L.A.V. Marlow

**Affiliations:** Cancer Research UK Health Behaviour Research Centre, Department of Epidemiology & Public Health, UCL, London, UK

**Keywords:** Head and neck cancer, HPV, human papillomavirus, knowledge, quality of life

## Abstract

Head and neck cancer (HNC) currently affects approximately 11 200 people in the UK, with an increasing proportion known to be caused by the human papillomavirus (HPV). We undertook a systematic review of studies measuring the psychosocial impact of HPV-related HNC and also studies measuring knowledge about the link between HPV and HNC among different populations. Searches were conducted on MEDLINE, Embase, PsycINFO, CINAHL Plus and Web of Science, with reference and forward citation searches also carried out on included studies. Studies were selected if they (i) were original peer-reviewed research (qualitative or quantitative), (ii) mentioned HPV and HNC, (iii) measured an aspect of the psychosocial impact of the diagnosis of HPV-related HNC as the dependent variable and/or (iv) measured knowledge of the association between HPV and HNC. In total, 51 papers met the inclusion criteria; 10 measuring psychosocial aspects and 41 measuring knowledge of the link between HPV and HNC. Quality of life in those with HPV-positive HNC was found to be higher, lower or equivalent to those with HPV-negative HNC. Longitudinal studies found quality of life in patients was at its lowest 2–3 months after diagnosis and some studies found quality of life almost returned to baseline levels after 12 months. Knowledge of the link between HPV and HNC was measured among different populations, with the lowest knowledge in the general population and highest in medical and dental professionals. Due to the limited studies carried out with patients measuring the psychosocial impact of a diagnosis of HPV-positive HNC, future work is needed with the partners of HPV-positive HNC patients and health professionals caring for these patients. The limited knowledge of the association between HPV and HNC among the general population also indicates the need for research to explore the information that these populations are receiving.

## Statement of Search Strategies Used and Sources of Information

We searched MEDLINE, Embase, PsycINFO, CINAHL Plus and Web of Science using relevant search terms for the overview. There were no language or date restrictions applied to the search. All references were reviewed against the inclusion and exclusion criteria. Additional relevant papers were found through searching the reference lists of included studies and carrying out forward citation searches on included studies.

## Introduction

Head and neck cancer (HNC) affects about 11 200 people in the UK each year [1], [2], [3], [4] and there are currently 62 500 survivors of the disease [5]. Diagnosis is associated with well-recognised psychosocial sequelae, including difficulties with communication with the partner, functioning in the family, and social and interpersonal relationships [6]. Patients have also been found to isolate themselves from their friends and family due to the disfiguring impact of treatment on their appearance [7], and many feel stigmatised [8]. Practical issues, such as problems with speaking, can also interfere with communication, and poorer communication has been found to result in greater distress [9]. Negative psychological consequences tend to be worse for patients undergoing surgery than those receiving alternative methods of treatment [10], [11], [12].

It is now clear that an increasing proportion of HNC cases are caused by the human papillomavirus (HPV) [13], [14], [15], [16], which has long been associated with cervical and other anogenital cancers [17]. The incidence of HPV-related HNC is rising [18], with numbers in the USA set to surpass the numbers of cervical cancer cases by 2020 if the current trend continues [19]. Patients with HPV-positive HNC are typically younger than those with HPV-negative disease, and tend to be white, male, married, educated and employed [20]. Risk factors are thought to be a greater lifetime number of sexual and oral sex partners [20], [21], [22], [23] due to greater exposure to HPV.

We know from the cervical cancer literature that the sexually transmitted nature of HPV can lead to additional psychological challenges to those associated with the cancer itself. Women have felt stigma, anxiety, concern about their relationships and worry about disclosure of their test results to others, following a diagnosis of HPV in the context of cervical screening [24]. As the link between HPV and HNC has been established, there has been increasing recognition of the need for guidance on how to discuss HPV with patients [25], [26], [27]. Behavioural and psychological science has made a significant contribution to understanding and addressing psychosocial issues associated with both HNC and HPV. One study measuring the supportive care needs of HNC patients found overwhelming evidence of unmet psychological needs [28] and a Cochrane review paper found a number of randomised controlled trials implementing psychosocial interventions for HNC patients, but concluded there was not enough evidence to conclude on their effectiveness [29]. It is also important to assess knowledge of the association between HPV and HNC among different populations, to identify gaps in knowledge and inform communication strategies.

Research has started to explore what the public know about HPV and HNC and how an HPV diagnosis affects patients. Knowledge of HPV appears to have increased following the introduction of the HPV vaccination, which is now offered to adolescents in most developed countries [30]. In the context of cervical cancer, an online survey of adults in the UK, USA and Australia following the introduction of the HPV vaccination showed 61% reported having heard of HPV [31]. By contrast, public awareness of the signs and risk factors for HNC has been shown to be poor [32]. In the clinical context, few resources currently exist to answer patients' concerns about how, when and why they got their cancer [27], the answers to which can have implications both for the patient and their past, present or future partners.

This review is timely in drawing together findings from the emerging literature and identifying priorities for a behavioural science research agenda in this field. The evidence from both the cervical cancer and HNC literature suggests that there may be greater psychological distress in these patients due to the combination of both a diagnosis of cancer and of HPV. The review aims to answer two questions:

(i) What is the psychological impact of an HPV diagnosis in the context of HNC?
(ii) What is known about HPV-related HNC in different population groups?

## Materials and Methods

### Search Methods for Identification of Studies

Preferred Reporting Items for Systematic Reviews and Meta-Analyses (PRISMA) guidelines were followed for this systematic review [33]. Search terms were developed in consultation with a librarian at UCL and through extracting key terms from previous review papers and relevant primary research. Terms covered (i) the health condition of interest (e.g. HPV, human papillomavirus, head and neck cancer), (ii) psychosocial outcomes of interest (e.g. psychosocial, depression, anxiety, quality of life) and (iii) knowledge (e.g. knowledge, awareness). The full search strategies for each database can be found in the Supplementary Material. Initial search terms were later refined based on common text words from relevant articles retrieved from the search. MEDLINE, Embase and PsycINFO databases were accessed through Ovid databases and were searched from inception to present in December 2014. Search terms were adapted for CINAHL Plus and Web of Science. These databases were chosen based on previous review papers in this field and because all databases will complement each other and allow a broader scope of coverage. There were no language or date restrictions applied to the search. The reference lists of included studies were searched (RD) for additional relevant papers. The grey literature was also searched using OPENSIGLE (opensigle.inist.fr). Results of the literature search were downloaded into Endnote with duplicate articles removed.

### Inclusion and Exclusion Criteria

Studies were included if they (i) were original peer-reviewed research (qualitative or quantitative), (ii) mentioned HPV and HNC, (iii) measured an aspect of the psychosocial impact of the diagnosis of HPV-related HNC as the dependent variable and/or (iv) measured knowledge of the association between HPV and HNC. Studies were excluded if they were not written in English, did not report original research or were conference abstracts.

### Selection Procedure and Quality Assessment

**Fig 1:**
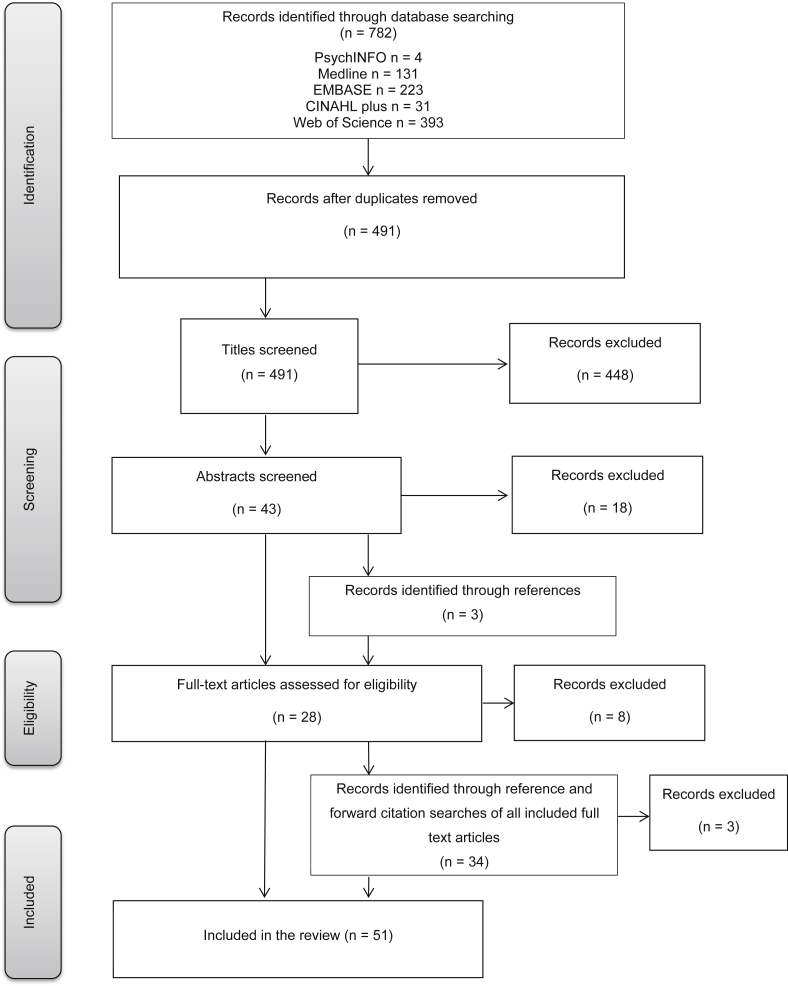
Flow diagram of study selection (adapted from [33]).

Article titles were screened by two authors (RD, JW) and were excluded if they were not written in the English language or were clearly irrelevant to the review. Two members of the review team (RD, LM) then screened the abstracts of the remaining articles, looking more specifically for articles mentioning HPV. Authors of conference abstracts that appeared to meet the eligibility criteria were contacted to request a copy of the full paper if it was available. Those not submitted or published in peer-review journals were excluded and one author did not reply after multiple attempts to contact them, so this paper was also excluded. Disagreements were resolved by discussion and reasons for inclusion/exclusion were noted. Articles that appeared to meet the inclusion criteria based on the title and abstract screen were obtained for full-text review (Figure 1). Copies of articles that could not be assessed for relevance based on the title and abstract screen were also obtained to determine eligibility based on full-text review.

A full-text eligibility review was conducted by two members of the review team (RD, LM) with reasons for exclusion recorded. Reasons for excluding studies included the article not containing any original data relevant to our eligibility criteria, not mentioning HPV or not measuring our outcomes of interest as dependent variables. Forward citation searches were conducted for all papers obtained for full-text review and included those published up to August 2015. Eligibility of articles found through forward citation and reference searching was confirmed by a second screener (LM). A summary of the data from all full-text articles was extracted (Table 1, Table 2) into Microsoft Excel and the quality of the studies was assessed using an amended version of the National Institute for Health and Care Excellence (NICE) quality appraisal checklists for quantitative and qualitative studies [34]. This considered a range of factors, which included whether the source population was well described, whether the outcome measures were reliable and relevant, whether the analytical methods were appropriate and whether the findings could be generalised to the source population.

### Analysis

Data from all included articles were recorded using a data extraction form. The results from articles measuring psychosocial outcomes and knowledge are reported descriptively with comparisons drawn where appropriate. Qualitative findings are described separately.

## Results

### Search Results

The initial search returned 782 articles, which was reduced to 491 after the removal of duplicates; 448 were excluded on the basis of their title, leaving 43 abstracts to be reviewed. Once the articles had been screened by title and abstract, 25 were obtained for full-text review. An additional 37 articles were included after searching the reference lists, relevant review papers found through the search and searching forward citations of those already obtained for full-text review. Eleven articles were excluded during full-text review, leaving 51 papers included in the final analysis. Figure 1 shows the study selection process. All the authors agreed on the final papers included in the review.

### Studies Assessing the Psychosocial Impact of Human Papillomavirus-related Head and Neck Cancer

Ten of the studies measured psychosocial outcomes [35], [36], [37], [38], [39], [40], [41], [42], [43], [44]. Of these, eight were conducted in the USA [35], [37], [38], [39], [40], [42], [43], [44], one was conducted in Switzerland [36] and one in Italy [41]. Quantitative studies used survey-based methods [36], [37], [38], [40], [41], [42], [43], [44] and conducted an audit on medical records [39]. One article collected qualitative data from individual interviews [35]. All articles were published between 2012 and 2014. In some studies, p16 expression was used as a marker of HPV status, but for simplicity we refer to patients as HPV-positive throughout the review.

The psychosocial impact of HPV-related HNC was measured in patients [35], [36], [37], [38], [39], [40], [41], [42], [43], [44] at different time points in their care continuum from newly diagnosed [36], [38], [40], [42], [43], to up to 5 years post treatment completion [35].

### Quality of Life Measures

Quality of life was the main outcome measure used in seven studies and was measured using a number of different tools. Six of the studies measuring quality of life used at least one HNC-specific measure (Table 3). Two studies used the Head and Neck Cancer Inventory (HNCI) [37], [38], which is a validated 30-item survey measuring patient-reported quality of life status in speech, eating, aesthetics and social disruption. Three studies used the University of Washington Quality of Life (UWQOL) measure [40], [43], [44], which is a validated HNC-specific quality of life questionnaire including 12 domains, with two subscales of physical and social-emotional functioning. One study used the European Organization for Research and Treatment of Cancer (EORTC) HNC-specific version (QLQ-H&N35) [36], which includes seven scales measuring pain, swallowing, senses, speech, social eating, social contact and sexuality. One study used Head and Neck Quality of Life (HNQOL) [44], which measures the four domains of eating, communication, pain and emotion. Generic measures were also used in three studies and included the Medical Outcome Study Short Form 36 (SF-36) [44], which is a 36-item generic measure of health status split into 10 domains, the EQ-5D [41], which has five dimensions of measuring quality of life, and the EORTC general core questionnaire (QLQ-C30) [36] measuring activity, physical and social functioning. Other psychosocial measures used were the National Comprehensive Cancer Network's distress thermometer, which uses a scale from 0 to 10 for patients to indicate how much distress they have been experiencing in the last week, and the Glinder and Compas one-item measure of behavioural blame [42] (i.e. ‘How much do you blame yourself for the kinds of things you did, that is, for any behaviours that may have led to your cancer?’).

### Studies with Human Papillomavirus-positive Patients Only

Three studies did not include a comparison between HPV-positive patients and HPV-negative patients [35], [42], [44]. The one qualitative study with HPV-positive HNC survivors reported that 3/10 cancer survivors felt a sense of stigma or shame associated with their diagnosis [35]. The second study measured distress and self-blame in newly diagnosed HPV-positive patients [42]. Distress levels were found to be moderate (mean 3.38, range 0–9), with 30% showing clinically meaningful scores (scores above or equal to 4). Self-blame levels were found to be low (mean 2.27, range 1–4). The third study measured quality of life using the UWQOL, HNQOL and SF-36 and found summary scores remained stable between 2 years and long-term follow-up (median of 78 months after the completion of treatment) [44]. Clinically meaningful (≥10 point change) declines in quality of life measured using the UWQOL was found in 14% of patients, whereas 11% of patients reported clinically meaningful improvements. Summary scores on this measure between pre-treatment and long-term follow-up were significantly worse. Clinically meaningful (≥10 point change) declines in quality of life measured using the HNQOL were found in 8% of patients, with 8% of patients reporting clinically meaningful improvements. Summary scores on this measure remained stable between pre-treatment and long-term follow-up. Scores on the SF-36 for long-term physical and mental health were comparable with the US population norms [44].

### Cross-sectional Studies with a Comparison Group

One study compared quality of life in HPV-positive patients and HPV-negative patients using the EORTC QLQ-C30 [36]. Patients with HPV-positive tumours were found to score significantly better on physical and role functions of the scale [36], but there were no significant differences between the groups in the emotional, social and global health functions of the scale. In an audit of medical records, Hess and colleagues [39] found that there was a higher prevalence of anxiety in HPV-negative patients, but rates of major depression or anxiety disorder did not differ significantly between the HPV-positive and HPV-negative groups. Another study compared quality of life scores measured using the EQ-5D between HPV-positive patients and healthy subjects [41]. Overall quality of life was significantly lower in patients than in healthy subjects.

### Longitudinal Studies with a Comparison Group

Four studies compared quality of life between HPV-positive and HPV-negative patients at more than one time point [37], [38], [40], [43]. One study measuring quality of life using the UWQOL [40], found that overall quality of life scores were better at each time point for HPV-positive patients than for HPV-negative patients, with the differences being significant at baseline, 6 months and after 12 months. Another study measuring quality of life using the UWQOL [43] found that pre-treatment quality of life scores were significantly higher in HPV-positive patients compared with HPV-negative patients, lower (but not significantly) immediately after treatment and similar at 1 year after treatment. Quality of life measured using the HNCI in one study found that HPV-positive patients had a higher quality of life at baseline, but then a lower quality of life at 3 weeks, 3 months and 6 months compared with HPV-negative patients [37]. Another study using the HNCI found that HPV status was not associated with quality of life outcomes at 12 months [38]. Using data from the whole sample, clinically meaningful declines were found from baseline to 12 months in speech function, aesthetic attitude, eating function and attitude [38].

Overall, these longitudinal studies found inconsistent results when comparing quality of life in HPV-positive patients and HPV-negative patients. Some reported HPV-positive patients with a combination of both higher and lower quality of life scores than HPV-negative patients depending on the time points (*n* = 3), with differences only significant when the quality of life scores were higher in HPV-positive patients. Others found no significant differences between the groups at any time point (*n* = 2).

### Studies Assessing Knowledge of Human Papillomavirus-related Head and Neck Cancer

Forty-one papers from 37 studies assessed knowledge about HPV and HNC [45], [46], [47], [48], [49], [50], [51], [52], [53], [54], [55], [56], [57], [58], [59], [60], [61], [62], [63], [64], [65], [66], [67], [68], [69], [70], [71], [72], [73], [74], [75], [76], [77], [78], [79], [80], [81], [82], [83], [84], [85]. Over half (*n* = 23) were conducted in the USA [46], [47], [48], [49], [52], [54], [56], [62], [67], [68], [69], [70], [71], [73], [74], [75], [76], [77], [78], [80], [82], [83], [84], [85], with others from Germany (*n* = 4) [58], [59], [60], [61], Saudi Arabia (*n* = 3) [63], [64], [65], Canada (*n* = 2) [50], [66], Malaysia (*n* = 2) [79], [81], Jordan (*n* = 2) [45], [57], Italy (*n* = 1) [72], Puerto Rico (*n* = 1) [51], Romania (*n* = 1) [55] and Ireland (*n* = 1) [53]. All were published between 2002 and 2015. Quantitative studies (*n* = 40) [45], [46], [47], [48], [49], [50], [51], [53], [54], [55], [56], [57], [58], [59], [60], [61], [62], [63], [64], [65], [66], [67], [68], [69], [70], [71], [72], [73], [74], [75], [76], [77], [78], [79], [80], [81], [82], [83], [84], [85] used survey-based data collection methods and one qualitative study collected data using focus groups [52].

Studies assessing knowledge of HPV and HNC included samples of dental students [48], [49], [55], [65], [81], medical students [64], [74], [81], [85], general undergraduate students [66], [82], [83], [85], oral health providers (dentists and dental hygienists) [46], [50], [52], [53], [58], [59], [60], [69], [71], [75], [79], head and neck surgeons [68], healthcare professionals [57], [61], [63], [75], a population-based sample of US men [47], [76] and a population-based sample of US adults [54], [67], [73], [78], [80]. Some specific sample populations were included, such as American Indian community members [56], bisexual and homosexual populations [62], [72], [77], [84] and National Association for Stock Car Auto Racing (NASCAR) fans [85].

Knowledge of the association between HPV and HNC varied across study populations and the questions asked (Table 4). All the questions involved recognition of HPV as either a cause or a risk factor for oral cancer, with no studies requiring participants to recall HPV as a risk factor for oral cancer. For example, Hertrampf and colleagues [58], [59], [60], [61] asked 'Which of the following factors places an individual at high risk for oral cancers?' with HPV listed as a response option and Cólon-López and colleagues [51] asked participants to respond true or false to the statement 'HPV is associated with oral cancer'. Knowledge of HPV as a risk factor for oral cancer ranged from 26 to 91% in medical or dental professional samples [45], [46], [50], [53], [57], [58], [59], [60], [61], [63], [68], [69], [71], [75], [79] compared with between 1 and 44% in samples of members of the general population [51], [54], [56], [60], [62], [67], [70], [72], [73], [76], [77], [80], [84], [47], [78]. Knowledge among students ranged from 18% in general undergraduate students to 84% in undergraduate dental students [48], [49], [55], [64], [65], [66], [74], [81], [82], [83].

### Quality Assessment

Based on the NICE quality appraisal checklists for the quantitative studies, 27 studies were designed or conducted in a way that minimised bias, nine studies were partly designed or conducted to minimise bias and had aspects of the study design that were unclear, and 13 studies were either unclear on aspects of the study reported or may not have addressed all potential sources of bias. No studies were assessed as having significant sources of bias across all aspects of the study design. Most studies described the source population well, used reliable and valid outcome measures, measured outcomes that were relevant and used appropriate analytical methods. Of 25 studies in which it was relevant to carry out a power calculation, only eight did so. Many of the studies had small samples and so could not be generalised to the source population. For the two qualitative studies, both were clear in the purpose of the study, carried out the data collection appropriately, were clear on the context in which the study was carried out, conducted reliable analysis, provided convincing findings and drew relevant conclusions. Both studies were unclear about whether the relationship between the researcher and participants had been considered. Both studies were considered to be designed to have minimised bias.

## Discussion

This review is the first to draw together the emerging literature on the psychosocial implications of an HPV-related HNC diagnosis and awareness of the link between HPV and HNC. Quality of life was measured in the HPV-related HNC patient population, with inconsistent results found. Quality of life in those with HPV-positive HNC was found to be higher, lower or equivalent to those with HPV-negative HNC. In longitudinal studies, irrespective of the instrument used, quality of life in patients was at its lowest 2–3 months after diagnosis. In some studies, quality of life almost returned to baseline levels after 12 months. The UWQOL was the instrument used in three of the 10 studies included in this review. This scale is specific to HNC and measures 12 different domains as single-item questions. To allow for comparisons across studies, it would be ideal to have a well-validated, standardised measure that could be used in all studies. As previously reported, it is difficult to make generalised statements about quality of life that can aid in clinical decision making, due to inconsistencies in the design of quality of life instruments for HNC and a lack of unified reporting standards [86].

Use of other psychosocial measures was limited, with only one other primary research study measuring domains other than quality of life. This one study found clinically meaningful levels of distress in 30% of patients, but relatively low levels of self-blame [42], suggesting there may be a need for interventions that may help alleviate distress levels. In the one qualitative study, a few survivors of HPV-positive HNC reported feelings of stigma and embarrassment about their diagnosis and this affected their sexual relationships, consistent with findings from the cervical cancer literature [24] and previous research with health professionals documenting concerns of HPV-positive HNC patients [87]. It is therefore difficult to draw conclusions based on the limited research that has been conducted around the psychosocial impact of HPV-related HNC. Future work is needed to explore the psychosocial impact of this diagnosis on the patient group, as well as their partners, the general population and health professionals.

The relationship between HPV and HNC is not well-known across most populations in the studies included here. The most knowledgeable group about HPV as a risk factor for HNC were second year dental students, dentists and head and neck surgeons (>85%), compared with one study finding that less than 1% of US adults knew that HPV is a risk factor for HNC. Awareness levels ranged across a variety of samples of the general population, dentists, students and specific sexually orientated groups, from 18 to 67%. Almost half the studies included dentists, dental hygienists or dental students, suggesting that the role dentists have to play in HPV and HNC is being increasingly recognised and educating them about HPV as a risk factor is important. All the questions were recognition questions rather than recall and so may not represent the true knowledge of participants as previous studies have found awareness to be higher in participants when responding to recognition questions when compared with recall [88], [89]. One study assessing knowledge in medical practitioners in Ireland found that when asked to list the risk factors they would associate with oral cancer, HPV was not listed [90]. There was also no standardised question assessing knowledge of the link between HPV and HNC, some asking it as a risk factor, whereas others were more specific (e.g. HPV is associated with some head and neck cancers). None of these studies were conducted in the UK, so no conclusions can be drawn about the level of knowledge in the UK. These studies were mainly from the USA, indicating a wide range of knowledge across different population subgroups, but that generally, there is a need for greater awareness.

### Strengths and Limitations

Adhering to PRISMA guidelines ensured this review was carried out systematically. By including quantitative and qualitative studies in the review, we avoided exclusion of any eligible and relevant studies. As a number of different instruments were used to measure quality of life and at different points in the patient care continuum, it was difficult to compare across studies.

## Conclusions

A limited number of studies have measured the psychosocial impact of a diagnosis of HPV-positive HNC and those few that have, have only measured this in patient populations. Future work is needed with the partners of HPV-positive HNC patients and health professionals caring for these patients. The limited knowledge of the association between HPV and HNC among the public also indicates the need for research to explore the information that these populations are receiving. The development of collaborations between behavioural scientists and clinicians in this field will help to ensure that awareness of the role of HPV in HNC is raised, and that the adverse psychological consequences associated with diagnosis are understood and minimised.

## Figures and Tables

**Table 1:** Psychosocial studies included in the systematic review

Reference Country	Sample	HPV/p16 positive	Study design	Outcomes/measures	Relevant findings
[35] USA	10 male survivors of HPV-OSCC between 1 and 5 years treatment completion	100% (HPV+)	Qualitative semi-structured interviews	Exploring the communication, comprehension and psychologic impact of a diagnosis of HPV-related oropharyngeal cancer	- 3/10 felt a sense of stigma or embarrassment associated with their diagnosis - The cancer itself occasionally or always overshadowed the impact of HPV - 4/10 were concerned with potentially infecting a partner with HPV and one expressed concerns about re-infection - Survivors understood and were encouraged by positive prognostic implications of an HPV diagnosis
[36] Switzerland	98 survivors of oropharyngeal cancer a median of 67 months after treatment	89% (p16+)	Follow-up survey (postal)	Quality of life: EORTC QLQ-C30 and EORTC QLQ-H&N35	p16– patients had significantly lower scores on physical and role functions and had more complaints about feeling ill and pain than p16+ patients
[37] USA	22 patients at first head and neck cancer clinic visit; 2 females, 20 males	80.9% (HPV+) 95.2% (p16+)	Longitudinal study: baseline (preoperatively), 3 weeks, 3 months, 6 months and 12 months	Quality of life: Head and Neck Cancer Inventory	- Speech, eating, aesthetics and social disruption scores at 3 weeks, 3 and 6 months were significantly lower than at baseline - Overall quality of life still significantly lower than preoperative levels at 1 year - Significant declines in overall quality of life at 3 months compared with 3 weeks - No significant impact on quality of life outcomes by HPV status
[38] USA	87 patients at first new patient referral visit: 81 included in analysis	63% (HPV+) 74% (p16+)	Prospective cohort study	Quality of life: Head and Neck Cancer Inventory	- All health-related quality of life scores declined at 3 weeks; social and overall scores continued to drop and bottomed out at 3 months - Social and overall scores showed at 12 months greatest recovery significantly from baseline - No differences between HPV+ and HPV– patients on any of the quality of life domains at 12 months
[39] USA	162 medical records – patients with locally advanced OSCC, known p16 status and treated by chemoradiation or primary surgery followed by adjuvant radiation therapy; 142 men, 20 women	69% (p16+)	Audit of medical records	Prevalence of anxiety disorder and major depression in patients with HPV+ and HPV– tumours	- No significant differences between HPV+ and HPV– patients for rates of major depression or anxiety disorder - Higher prevalence of anxiety in HPV– patients
[40] USA	177 patients with HNSCC and known HPV/p16 status diagnosed between 2006 and 2012	45% (p16+)	Longitudinal study: baseline, 2 months, 6 months, 1 year and 1–3 years	Quality of life: UWQOL	- p16+ patients had significantly better overall quality of life, recreation and chewing scores at baseline - p16+ patients had better activity, recreation, overall quality of life at 6 months - No long-term differences in quality of life for p16– patients treated with primary surgery or chemoradiation
[41] Italy	79 patients with HNSCC; 17 women, 62 men Control group: healthy subjects matched for gender and disease proportion attending same clinic for non-pathological reasons	100% (HPV+)	Multicentre, observational and retrospective study	Perception of their health conditions: EQ-5D Measurement of utility loss considering patient's perspective	- Overall quality of life in patients significantly lower than healthy subjects - Utility scores were higher in men than women
[42] USA	62 newly diagnosed HPV+ patients initiating radiotherapy	98% (p16+) 89% (HPV+)	Cross-sectional survey	Self-reported: - Feelings of keeping their HPV a secret from others - Disclosure of HPV to current sexual partner - Whether HPV increased partner's risk - Whether they talked to partner about likelihood of transmission - How much knowledge of HPV as a cause had impacted their relationship - Distress - Self-blame	- About 30% showed marked distress - Distress levels were moderate - Patients reported low levels of behavioural self-blame - Blame and distress were significantly correlated - No significant differences regarding distress and self-blame in those self-declaring as HPV+ compared with those who did not or were unsure - 14% intended to keep it a secret from others and 3% did not tell their partner – reasons included embarrassment, stigma, and belief it is no-one else's business - 41% said they had not discussed concerns regarding potential viral transmission to their partner - 8% thought their HPV had entirely increased their partners risk for developing cancer, 42% said somewhat and 29% said it did not - 80% reported that the diagnosis had no negative impact, 14% reported a somewhat negative impact, 6% reported a completely negative impact on relationships
[43] USA	228 patients diagnosed with primary OSCC between 2003 and 2010 Group 1: (*n* = 162) HPV– and low-risk HPV Group 2: (*n* = 66) High-risk HPV+	29% (HPV+)	Longitudinal study: pre-treatment, immediate post-treatment and 1 year post-treatment	Quality of life: UWQOL	- Pre-treatment quality of life scores were significantly higher in patients who were high-risk HPV+ - Immediate post-treatment scores were lower in HPV+ patients - Post-treatment scores were similar between the two groups - Group 2 had a significantly larger decrease in quality of life scores from pre-treatment to immediate post-treatment compared with group 1 - The change in quality of life scores from post-treatment to 1 year post-treatment was similar between the groups - HPV status was associated with pre-treatment quality of life and a change in quality of life from pre-treatment to immediate post-treatment - Patients in group 2 did not have better 1 year quality of life compared with group 1
[44] USA	40 head and neck cancer survivors >2 years after treatment: 34 men, 6 women	98% (HPV+)	Follow-up survey (postal)	Health-related quality of life: HNQOL, UWQOL, SF-36	- Global HNQOL remained stable compared with 2 year assessments for HNQOL and UWQOL - Clinically meaningful declines in global HRQOL from 2 year assessment were reported by 8% of patients by HNQOL and 14% of patients by UWQOL - 8% on HNQOL and 11% on UWQOL reported meaningful improvements in global HNQOL - 84% and 75% of patients reported stable global HRQOL compared with 2 years by HNQOL and UWQOL - Overall physical and mental health mean scores on the SF-36 were comparable to US population norms in each HRQOL domain - Overall cohort experienced stable HNQOL scores and statistically worse UWQOL score compared to pre-treatment - Clinically meaningful declines were found in global HRQOL from pre-treatment by 8% on HNQOL and 30% on UWQOL

HPV, human papillomavirus; HNSCC, head and neck squamous cell carcinoma; EORTC, European Organization for Research and Treatment of Cancer; UWQOL, University of Washington Quality of Life; HNQOL, Head and Neck Quality of Life; SF-36, Short Form 36; OSCC, Oropharyngeal squamous cell carcinoma.

**Table 2:** Included studies measuring knowledge of human papillomavirus (HPV) and head and neck cancer

Reference Country	Sample	Response rate	Study design	Outcomes/measures	Relevant findings
[45] Jordan	112 newly graduated medical and dental SHO level; 49% dental degree, 51% medical	Not reported	Survey (in person)	Knowledge of risk factors for oral cancer (e.g. Which of the following factors is considered an increased factor for oral cancer: Human papillomavirus as a response option)	- HPV correctly identified as a risk factor by 34% - more dental (47% versus 21%) than medical responded correctly
[46] USA	651 dental hygienists from North Carolina State Board of Dental Examiners	53%	Survey (postal)	Knowledge of risk factors for oral cancer (e.g. In the United States, which of the following factors places an individual at high risk for oral cancer? Human papillomavirus as yes/no/don't know option)	- 47.1% knew HPV a risk factor for oral cancer - 32% felt patients are knowledgeable about oral cancer risk factors
[47]∗ USA	609 men aged 18–59 years from population-based panel of US households: Men's Health Study	70%	Survey (online)	Awareness and knowledge (e.g. Which of the following do you think might increase the chances of getting oral cancer? Infection with a virus as a response option) Beliefs about causes of HPV-related disease (e.g. Do you think HPV can cause oral cancer? Yes/no/don't know)	- More men knew HPV can cause genital warts (41%) than oral cancer (23%) - 43% identified infection with a virus as a potential cause of oral cancer (less than for anal cancer or genital warts) - Few believed sexual behaviours increases risk of oral cancer (23% having sex; 26% high number of sexual partners)
[48] USA	248 1st, 2nd, 3rd and 4th year dental students at University of Maryland Baltimore College of Dental Surgery	59.6%	Cross-sectional survey (in person and postal)	Knowledge of oral cancer risk factors	- 30.8% 1st year, 89.1% 2nd year, 78.1% 3rd year, 81.8% 4th year knew HPV is a risk factor for oral cancer
[49] USA	163 dental students, Medical University of South Carolina	79.1%	Survey (in person)	Knowledge of oral cancer risk factors	- 79.8% correctly identified HPV as a risk factor
[50] Canada	670 dentists, British Columbia and Nova Scotia	55.2%	Survey (postal)	Knowledge of oral cancer risk factors	- 53.1% correctly identified HPV as a risk factor for oral cancer
[51] Puerto Rico	206 Men in sexually transmitted disease clinic	Not reported	Survey (in person)	HPV awareness, HPV knowledge (e.g. HPV is associated with oral cancer: true/false/don't know)	- 27.4% knew HPV infection has a role in oral cancer
[52] USA	17 dentists in 2 focus groups, 21 dental hygienists in 2 focus groups	Not reported	Qualitative focus groups	Assess awareness of oral health providers regarding the HPV-oral cancer link Elicit attitudes and perceived role in screening for HPV-oral cancer lesions and discussing HPV as a contributing factor for oral cancer	- Participants ranged from a complete lack of knowledge to understanding some intricacies of the HPV-oral cancer link - Shifts in dentistry practice were seen as a result of the HPV-oral cancer link and there was a desire for additional guidance from professional organisation on ways to manage screening for HPV-related oral cancer - Discomfort was expressed in discussing the HPV-oral cancer link with patients, with concerns about the appropriateness of HPV-oral cancer discussions with patients due to confidentiality and gender roles - Responses varied as to whether it was their role to discuss with patients
[53] Ireland	254 dentists	Not reported	Cross-sectional survey (online)	Knowledge of oral cancer risk factors	- 60% knew HPV is a risk factor for oral cancer
[54] USA	93 community members	32%	Survey (telephone)	Knowledge of oral cancer risk factors	- 34% knew having HPV 'increases the risk of getting mouth or throat cancer'
[55] Romania	192 1st–6th year dental students; 139 female, 53 male	100%	Cross-sectional survey (in person)	Knowledge of oral cancer risk factors	- Almost 54% identified HPV as a risk factor for oral cancer
[56] USA	205 American Indian community members recruited via two community events; 70% female	Not reported	Survey (in person)	Knowledge of the risk factors of head and neck cancer including HPV (e.g. Do you think that HPV can cause head and neck cancer? yes/no/don't know)	- 32% had heard of head and neck cancer - 23% identified having multiple sexual partners as a risk factor - 36% thought HPV is related to head and neck cancer
[57] Jordan	330 primary healthcare professionals	87%	Survey (face-to-face interview)	Knowledge of oral cancer risk factors	- 43.3% identified Human papillomavirus as a risk factor
[58]† Germany	306 dentists in Schleswig-Holstein	14%	Survey (postal)	Knowledge of oral cancer risk factors (e.g. Which of the following factors places an individual at high risk for oral cancers? Human papillomavirus as yes/no/don't know option)	- 57.8% identified Human papillomavirus as a risk factor
[59] Germany	394 dentists in Schleswig-Holstein	17%	Survey (postal)	Knowledge of oral cancer risk factors (e.g. Which of the following factors places an individual at high risk for oral cancers? Human papillomavirus as yes/no/don't know option)	- 61.2% identified Human papillomavirus as a risk factor; 63.4% in those participating at re-evaluation and attending a continuing education course on oral cancer
[60]† Germany	306 dentists in Schleswig-Holstein; 1000 members of the public	14%	Survey (postal and telephone)	Knowledge of oral cancer risk factors (e.g. Which of the following factors places an individual at high risk for oral cancers? Human papillomavirus as yes/no/don't know option)	- 57.8% of dentists and 29% of the public identified Human papillomavirus as a risk factor
[61] Germany	388 medical practitioners in Schleswig-Holstein	13%	Survey (postal)	Knowledge of oral cancer risk factors (e.g. Which of the following factors places an individual at high risk for oral cancers? Human papillomavirus as yes/no/don't know option)	- Human papillomavirus recognised as risk factor by 70% otorhinolayngology, 54% GPs, 50% internal medicine (continuing education for general medical care), 51% internal medicine, 82% dermatologists
[62]∗ USA	609 men: 312 gay and bisexual, 296 heterosexual	70%	Survey (online)	Knowledge of HPV (e.g. Do you think HPV can cause oral cancer? Yes/no/don't know)	- 21% of heterosexual men and 25% of gay/bisexual men knew HPV can cause oral cancer
[63] Saudi Arabia	236 healthcare professionals	Not reported	Cross-sectional survey	Knowledge of oral cancer risk factors	- 39.1% knew Human papillomavirus is a risk factor for oral cancer
[64] Saudi Arabia	167 undergraduate medical students (all students in years 4–6)	100%	Cross-sectional survey (in person)	Knowledge of oral cancer risk factors (e.g. Which of the following factors places an individual at high risk for oral cancers? Human papillomavirus as yes/no/don't know option)	- 65.7% overall identified Human papillomavirus as high-risk factor of oral cancer - Male 4th year 19%; 5th year 17%, 6th year 16% - Female 4th year 5%, 5th year 4%, 6th year 4%
[65] Saudi Arabia	479 undergraduate dental students (all students in years 4–6)	87.1%	Cross-sectional survey (in person)	Knowledge of oral cancer risk factors	- 83.7% identified Human papillomavirus as placing someone at high risk for oral cancer - Male 4th year 10%; 5th year 15%; 6th year 15% - Female 4th year 12%; 5th year 15%; 6th year 16%
[66] Canada	176 males at postsecondary institutions in Greater Vancouver	Not reported	Survey (in person)	Knowledge of HPV	- 32.9% knew 'HPV infections can cause oral cancers' and 24.2% knew 'HPV infections can cause pharyngeal (throat) cancers'
[67] USA	2126 US adults from Harris Interactive online panel	Not reported	Cross-sectional survey (online)	Awareness (e.g. Did you know that the virus HPV (human papillomavirus) that causes cervical cancer is also associated with throat cancer?) Knowledge (e.g. How knowledgeable are you about oral, head, and neck cancer? Likert scale not at all to extremely knowledgeable)	- 66% considered themselves not very or not at all knowledgeable about head and neck cancer - Knowledge of HPV as a risk factor in 0.8% - 12.8% were aware of this association when specifically queried about the association between HPV and throat cancer - Respondents with a college or university degree were more likely to associate HPV with throat cancer (14.8% versus 10%) - Older age was associated with less knowledge of HPV as a risk factor
[68] USA	297 American Head and Neck Society head and neck surgeons	27.5%	Survey (online)	Assess clinical practices Assess attitudes Assess knowledge regarding HPV-related cancer of the head and neck	- 90.9% said they discuss HPV as a risk factor with patients - Respondents specifically with daughters - about 85% discussed HPV as a risk factor - Scored very well on knowledge items of HPV - in 5 out of 7, over 92% of responses were correct
[69] USA	619 dentists in Maryland	53.6%	Survey (postal)	Knowledge of oral cancer risk factors	- 88% knew HPV is a risk factor for oral cancer
[70] USA	303 drag racers (28.3%) and fans (70%), vendors (1.7%) attending annual United Black Drag Racers drag racing event in St Louis	Not reported	Survey (in person)	Knowledge of HPV and head and neck cancer (e.g. Please indicate whether you think that each of these things may or may not increase a person's chance of getting head and neck cancer: Human papillomavirus infection; certain types of HPV can lead to oral cancer: True)	- 29.9% knew HPV definitely increases the risk of developing oral, head and neck cancer - Male 49%; Female 62.6%
[71] USA	584 licensed dentists in North Carolina	52%	Survey (postal)	Knowledge of oral cancer risk factors (e.g. In the United States, which of the following factors places an individual at high risk for oral cancer? Human papillomavirus listed as an option)	- 60% aware of Human papillomavirus as a risk factor
[72] Italy	1000 lesbian, gay and bisexual men and women	86.8%	Cross-sectional survey (in person)	Know that HPV can cause oropharyngeal cancer	- 47% gay men, 44% lesbians, 31% bisexual men and 35% of bisexual women knew oral cancer is an HPV-related disease - The vast majority knew unprotected sex was the main risk factor - 60.6% had heard of HPV
[73] USA	62 senior citizens	66%	Survey (in person)	Knowledge of oral cancer risk factors	- 29.5% knew infection with HPV was a contributing factor for oral cancer
[74] USA	450 medical students, South Carolina	78.8%	Cross-sectional survey (in person)	Knowledge of oral cancer risk factors	- 61.4% overall knew HPV is associated with an increased risk for oral cancer; 33.7% 1st year; 58.7% 2nd year; 80.8% 3rd year; 64.7% 4th year
[75] USA	269 dentists, 19 oral surgeons, 221 physicians	57% dentists 76% oral surgeons 45% physicians	Cross-sectional survey (postal)	Knowledge of oral cancer risk factors (e.g. Rank (high, medium, low) the association of known high-risk factors (Human papillomavirus) with oral cancer)	- Human papillomavirus ranked as high risk by 26% dentists, 37% physicians; medium risk by 49% dentists and 45% physicians; low risk by 26% dentists, 37% physicians
[76]∗ USA	609 men aged 18–59 from national panel of US households	70%	Cross-sectional survey (online)	Knowledge of HPV (e.g. Do you think HPV can cause oral cancer? Yes/no/don't know)	- 21% of those having heard of HPV responded yes to HPV can cause oral cancer
[77]∗ USA	306 men self-identified as gay or bisexual aged 18–59 from national panel of US households	70%	Cross-sectional survey (online)	Knowledge of HPV (e.g. Do you think HPV can cause oral cancer? Yes/no/don't know)	- 25% of those having heard of HPV responded yes to HPV can cause oral cancer
[78] USA	2393 general population from rural areas	Not reported	Survey (telephone)	Knowledge of risk factors for mouth and throat cancer	- 40.2% Having Human papillomavirus 'Yes-it increases the risk of getting mouth or throat cancer'
[79] Malaysia	362 dentists	41.7%	Survey (in person)	Knowledge of oral cancer risk factors	- 67.2% knew Human papillomavirus is a risk factor for oral cancer
[80] USA	267 parents of sons eligible to receive HPV vaccination	Not reported	Cross-sectional survey (in person)	Parents' knowledge of HPV in oropharyngeal cancer	- 18% knew role of HPV in oropharyngeal cancer
[81] Malaysia	147 final year medical and dental undergraduates of Universiti Sains Malaysia	73.5%	Survey (in person)	Aetiology of oral cancer	- 59.6% of medical students and 75.6% of dental students knew of role of HPV in aetiology of oral cancer (not statistically significant difference)
[82] USA	68 male African American college students, St Louis	Not reported	Cross-sectional survey (online)	Knowledge of HPV	- 60.2% knew HPV can cause oral cancer in men; 61.7% knew HPV can cause oral cancer in women
[83] USA	361 freshman students at Texas State University	10.7%	Survey (online)	Knowledge: - HPV can be contracted through oral sex - HPV has a strong correlation with oropharyngeal cancer - HPV is associated with some head and neck cancers	- 71.5% knew HPV could be contracted through oral sex - 51.6% knew of an association between HPV and oropharyngeal cancer - 18.2% knew HPV is associated with some head and neck cancers
[84] USA	179 men self-identified as gay and bisexual aged 18–29 from student organisations and social networking sites	Not reported	Survey (online)	Knowledge of HPV	- 25% of those having heard of HPV responded yes to HPV can cause oral cancer
[85] USA	491 NASCAR fans, 158 medical students, 186 undergraduate students	Not reported	Survey (in person)	Awareness of relationship between HPV and head and neck cancer (e.g. How much do you agree that HPV increases the risk of head and neck cancer?)	- Mean score: Medical students 2.84; Undergraduates 2.31; NASCAR 2.63

∗ These four papers used the data from one study.

† These two papers used the data from one study.

**Table 3:** Scores from psychosocial measures in human papillomavirus (HPV)-related head and neck patients in nine∗ studies

Reference	Measure	HPV+	HPV–	Significant difference
[36]	Quality of life - EORTC QLQ-C30 (median score; scale range 0–100)			
Emotional	91.67	83.33	NS
Social	100	100	NS
Global health	83.33	79.17	NS
[37]	Quality of life - HNCI (mean score; scale range 0–100)			
Baseline	94	75	NS
3 weeks	79	88	NS
3 months	48	58	NS
6 months	63	83	NS
12 months	88	/	NS
[38]	Quality of life - HNCI (mean score; scale range 0–100)			
12 months	75	78	NS
[40]	Quality of life - UWQOL (mean score across 12 domains; scale range 0–100)			
Baseline	76	50	0.008
2 months	57	51	NS
6 months	67	59	0.034
12 months	69	64	NS
>12 months	82	65	0.013
[43]	Quality of life - UWQOL (mean score across 12 domains; scale range 0–100)			
Pre-treatment	86	79	0.015
Immediate post-treatment	63	73	NS
Post-treatment	75	77	NS
[39]	Major depression	9%	10%	NS
Anxiety disorder	6%	12%	NS
[42]	Distress (mean; scale range)	3.38 (0–9)	/	
Self-blame (mean; scale range)	2.27 (1–4)	/
[41]	EQ-5D (mean utility values)			
Women	0.7	/	
Men	0.8	/
[44]	Quality of life - UWQOL (mean score; scale range 0–100)			
	Pre-treatment	10	/	
	24 months	15.2	/	
	Long term	16.5	/	
	HNQOL (mean score; scale range 0–100)			
	Pre-treatment	15.1	/	
	24 months	9.5	/	
	Long term	11.9	/	

EORTC, European Organization for Research and Treatment of Cancer; HNCI, Head and Neck Cancer Inventory; UWQOL, University of Washington Quality of Life; HNQOL, Head and Neck Quality of Life.

∗ One reference not included as used qualitative methodology [35].

**Table 4:** Knowledge about human papillomavirus (HPV) and oral cancer reported in 35∗ studies

Question	% (reference)	Sample population
Heard of HPV … (closed question)	70.6% [47]	General population men (USA)
61% [76]	General population men (USA)
79% [77]	General population men (USA)
93% [84]	General population men (USA)
60.6% [72]	General population (Italy)
59% [56]	General population (USA)
80% [66]	College students (Canada)
85% [82]	College students (USA)
HPV as a risk factor for oral cancer was known by … (closed question)	53.1% [50]	Dentists (Canada)
60% [71]	Dentists (USA)
57.8% [58]	Dentists (Germany)
26% [75]	Dentists (USA)
61.2% [59]	Dentists (Germany)
60% [53]	Dentists (Ireland)
57.8% [60]	Dentists (Germany)
88% [69]	Dentists (USA)
67.2% [79]	Dentists (Malaysia)
47.1% [46]	Dental hygienists (USA)
79.8% [49]	Dental students (USA)
66.5% [48]	Dental students (USA)
54% [55]	Dental students (Romania)
83.7% [65]	Dental students (Saudi Arabia)
34% [45]	Newly graduated medical and dental personnel (Jordan)
37% [75]	Physicians (USA)
39.1% [63]	Healthcare professionals (Saudi Arabia)
91% [68]	Head and neck surgeons (USA)
50–82% [61]	Medical practitioners (Germany)
43.3% [57]	Healthcare professionals (Jordan)
61.4% [74]	Medical students (USA)
65.7% [64]	Medical students (Saudi Arabia)
44% [72]	General population (Italy)
29% [60]	General population (Germany)
32% [54]	General population (USA)
40.2% [78]	General population (USA)
0.8% [67]	General population (USA)
29.5% [73]	General population (USA)
29.9% [70]	General population (USA)
18% [80]	General population (USA)
Knew HPV can cause oral cancer/head and neck cancer	23.3% [47]	General population men (USA)
21% [76]	General population (USA)
25% [77]	General population (USA)
39% [84]	General population men (USA)
21–25% [62]	General population men (USA)
27.4% [51]	General population men (Puerto Rico)
36% [56]	General population (USA)
12.8% [67]	General population (USA)
18.2% [83]	College students (USA)
59.6% [81]	Medical Students (Malaysia)
75.6% [81]	Dental Students (Malaysia)
32.9% [66]	College students (Canada)
60.2% in men [82]	College students (USA)
61.7% in women [82]	College students (USA)
Knew HPV is strongly associated with oropharyngeal cancer	51.6% [83]	College students (USA)

∗ Two references not included as the studies did not use comparable methodology [52], [85].
