# Supplementary material for: Human Papillomavirus and Head and Neck Cancer: Psychosocial Impact in Patients and Knowledge of the Link – A Systematic Review
Source: Clin Oncol (R Coll Radiol). 2016 Jul;28(7):421–39. doi: 10.1016/j.clon.2016.02.012 (PMC4914608; doi:10.1016/j.clon.2016.02.012)
Supplement: Supplementary file 1 [file mmc1.docx]

|  | **MEDLINE, EMBASE, PsycINFO** | **Web of Science** | **CINAHL plus** |
| --- | --- | --- | --- |
| Health condition of interest | 1] “Head and Neck Neoplasms”/ | ( oropharyngeal cancer* **OR** oropharyngeal neoplas* **OR** head AND neck cancer* **OR** head AND neck tumor **OR** head AND neck tumour **OR** head AND neck neoplasms) | 1] Oropharyngeal cancer* |
|  | 2] “Head and neck cancer”.mp.” |  | 2] “oropharyngeal neoplasms” |
|  | 3] “Head and neck tumor$”.mp. |  | 3] “head and neck cancer” |
|  | 4] “Head and neck tumour$”.mp |  | 4] (MH “Head and Neck neoplasms”) OR “head and neck neoplasms” |
|  | 5] Oropharyngeal Neoplasms/ |  |  |
|  | 6] Oropharyngeal cancer$.mp |  |  |
|  | 8] HPV.mp | ( hpv **OR** human papillomavirus **OR** human papilloma virus **OR** papillomavirus infections ) | 6] Hpv |
|  | 9] Human papillomavirus.mp |  | 7] Human papillomavirus |
|  | 10] Human papilloma virus.mp. |  | 8] Human papilloma virus |
|  | 11] Papillomavirus Infections/ |  | 9] Papillomavirus infections |
| Psychosocial outcomes of interest | 13] Psych$.mp. | ( psych* **OR** psychosocial **OR** depression **OR** anxiety **OR** "quality of life" **OR** knowledge) | 11] Psych* |
|  | 14] Depression/ or depression.mp |  | 12] Psychosocial |
|  | 15] Anxiety/ or anxiety.mp. |  | 13] MH Depression |
|  | 16] “Quality of life”/ or “quality of life.mp. |  | 14] MH Anxiety |
|  | 17] Psychosocial.mp |  | 15] MH Psychosocial aspects of Illness |
|  |  |  | 16] MH Psychology |
|  |  |  | 17] MH Quality of Life |
| Knowledge | 18] Knowledge/ or knowledge.mp |  | 18] (MH “knowledge”) OR “knowledge” |
| Search combinations | 1 or 2 or 3 or 4 or 5 or 6 |  | 1 or 2 or 3 or 4 |
|  | 8 or 9 or 10 or 11 |  | 6 or 7 or 8 or 9 |
|  | 13 or 14 or 15 or 16 or 17 or 18 |  | 11 or 12 or 13 or 14 or 15 or 16 or 17 or 18 |
|  | 7 and 12 and 19 |  | 5 and 10 and 19 |
| Search results | PsycINFO 4  MEDLINE 131  EMBASE 223 | WEB OF SCIENCE 393 | CINAHL PLUS 31 |

**Supplementary material: Search terms used for each database**
